# Incidence and characteristics of invasive fungal diseases in allogeneic hematopoietic stem cell transplant recipients: a retrospective cohort study

**DOI:** 10.1186/s12879-015-1329-6

**Published:** 2015-12-29

**Authors:** Nicole Harrison, Margit Mitterbauer, Selma Tobudic, Peter Kalhs, Werner Rabitsch, Hildegard Greinix, Heinz Burgmann, Birgit Willinger, Elisabeth Presterl, Christina Forstner

**Affiliations:** Department of Medicine I, Division of Infectious Diseases and Tropical Medicine, Medical University of Vienna, Währinger Gürtel 18-20, 1090 Vienna, Austria; Department of Medicine I, Bone Marrow Transplantation, Medical University of Vienna, Vienna, Austria; Division of Hematology, Medical University of Graz, Graz, Austria; Department of Laboratory Medicine, Division of Clinical Microbiology, Medical University of Vienna, Vienna, Austria; Department of Hospital Hygiene and Infection Control, Medical University of Vienna, Vienna, Austria; Center of Infectious Diseases, Jena University Hospital, Jena, Germany

**Keywords:** Invasive fungal disease, Candidiasis, Aspergillosis, Hematopoietic stem cell transplantation, Immunosuppression

## Abstract

**Background:**

Allogeneic hematopoietic stem cell transplant (HSCT) recipients experience an increased risk for invasive fungal diseases (IFDs).

**Methods:**

This retrospective cohort study at the Medical University of Vienna aspired to assess the incidence, characteristics and the outcome of IFDs as well as the associated risk factors in a setting where only 43 % of patients were given systemic antifungal prophylaxis during aplasia. IFDs were classified as probable or proven according to the EORTC/MSG consensus group. All adult patients (*n* = 242) receiving an allogeneic HSCT at the University Hospital of Vienna from January 2009 to December 2013 were enrolled.

**Results:**

The primary outcome of this study was the one-year incidence for IFDs after HSCT, which was 10.3 % (25/242). Overall 28 patients experienced an IFD – 20 probable and 8 proven – with invasive aspergillosis being the predominant IFD (*n* = 18), followed by invasive candidiasis (*n* = 7) and pneumocystis pneumonia (*n* = 3). Patients with an IFD were more likely to be admitted to an intensive care unit (64 % versus 12 %, *p* < 0.0001) and had a significantly higher mortality in the first year after HSCT (48 % versus 25 %, *p* = 0.02). Multivariate regression analysis revealed that intensified immunosuppressive therapy (high-dose cortisone and basiliximab or etanercept) because of severe graft-versus-host disease (adjusted odds ratio (AOR) 3.6, *p* = 0.01) and transplant-associated microangiopathy (AOR 3.7, *p* = 0.04) were associated with an increased risk for IFD, while antifungal prophylaxis given during aplasia and post-engraftment was associated with a decreased risk (AOR 0.3, *p* = 0.02).

**Conclusions:**

We documented a one-year incidence for IFDs of 10.3 % and no selection of rare pathogens at a centre with moderate use of antifungal prophylaxis. Intensified immunosuppressive therapy and transplant-associated microangiopathy were significant risk factors for IFDs.

## Background

Allogeneic hematopoietic stem cell transplant (HSCT) recipients experience an increased risk for opportunistic fungal infections, not only during aplasia, but for an extended period of time, often enhanced by certain risk factors such as severe graft-versus-host disease (GvHD) [1, 2]. Invasive fungal diseases (IFDs) still cause a considerably high mortality among these patients [3], despite the development of new antifungal agents for prophylaxis and treatment [4–7]. Their epidemiology continues to evolve as new transplantation strategies are implemented and the use of prophylaxis against fungal infections is broadened. The prophylactic use of fluconazole has decreased the incidence of invasive *Candida* infections [8, 9] placing stronger emphasis on the not yet declining mould infections [3].

As the use of antifungal prophylaxis is being increased, it becomes more important to define those patients most at risk for IFDs. Several risk factors for IFDs have been described, like HLA-mismatched donors, use of cord blood as graft, prolonged severe neutropenia, cytomegalovirus reactivation, severe GvHD, iron overload, intensified immunosuppressive treatment e.g. with high-dose steroids, and genetic risk factors such as toll-like receptor polymorphisms [2, 10–16].

To evaluate the epidemiology of IFDs and its ongoing changes including the use of antifungal prophylaxis, this retrospective cohort study at the Medical University of Vienna aspired to assess the incidence, characteristics and the outcome of IFDs as well as the associated risk factors.

## Methods

### Study population and data collection

Ethical approval for this study was obtained from the local Ethics Committee of the Medical University of Vienna (No. 982/2011). All adult patients receiving an allogeneic HSCT at the Bone Marrow Transplant Unit of the University Hospital of Vienna from January 2009 to December 2013 were enrolled. Their medical charts were reviewed retrospectively and follow-up data including information on mortality was obtained in January 2015. For those patients who received multiple transplants during the study period, only the most recent HSCT was included in the analysis. The patients’ demographic characteristics, details about the transplantation including donor, type of graft, conditioning regimen, and the post-transplant follow-up including the severity of GvHD [17], post-transplant complications as well as the use of prophylaxis and antifungal therapy were entered into a database. The transplantation risk for each patient was calculated using the European Society for Blood and Marrow Transplantation (EBMT) risk score which includes age at HSCT, disease stage, time from diagnosis to transplantation, donor type and donor recipient sex combination [18].

### Transplant details

Patients received peripheral blood stem cells (PBSC), bone marrow or cord blood as graft from HLA-matched related donors, matched unrelated donors (12/12 alleles) or mismatched unrelated donors (11/12 alleles). The myeloablative regimen consisted of cyclophosphamide and total body irradiation, while several different reduced-intensity conditioning (RIC) regimens were used. Acute GvHD prophylaxis included cyclosporine A (CSA) and methotrexate after myeloablative conditioning and CSA and mycophenolate mofetil after RIC. All patients were isolated in negative pressure rooms until sufficient engraftment was achieved. Systemic antifungal prophylaxis was not used generally for all patients during aplasia, but according to their risk profile (e.g. previous IFD) and to the attending clinical physician’s evaluation. Patients who developed fever during aplasia were treated empirically with antifungals if the fever persisted for more than 48 h despite antibiotic therapy.

### Screening and diagnostic procedures for IFDs

All patients were screened for invasive aspergillosis using serum galactomannan before HSCT and twice weekly after HSCT until they were discharged. During the follow-up patients were screened with serum galactomannan every two weeks until day +100. In case of fever the standard procedures included blood cultures at two different time points and a multiplex PCR which is capable to detect five different *Candida species* and *Aspergillus fumigatus*. Patients presenting with respiratory symptoms were first examined by chest X-ray and in case of infiltrates or worsening gas exchange followed-up with a computed tomography (CT) scan of the lungs. If the CT scan showed pulmonary infiltrates, bronchoscopy was performed and fungal culture, galactomannan and PCR for fungal pathogens including *Pneumocystis jirovecii* were conducted from the bronchoalveolar lavage fluid [19]. Transbronchial biopsy during bronchoscopy or CT-guided biopsy of suspected liver lesions was performed in some cases. Patients with neurological symptoms received magnetic resonance imaging (MRI) of the brain and lumbar puncture including culture and PCR for different fungal pathogens.

### Identification of fungal pathogens

All microbiological specimens were analysed for fungal pathogens by the department of clinical microbiology of the Medical University of Vienna. Blood cultures were incubated in the BacTAlert® (BioMerieux, France) for fourteen days and if a positive signal was obtained, a gram stain and a subculture on Sabouraud Glucose Agar (SAB) and Chromagar Candida® (Becton Dickinson, Heidelberg, Germany) were performed. Further identification of yeasts was done using either the Vitek® system or ATB Candida ID32C® (both BioMerieux, France). Also, rice-extract agar was inoculated and incubated at 28 °C for the formation and examination of micromorphology. Other specimens such as sputum or bronchoalveolar lavage fluid were inoculated on Sabouraud Glucose Agar (SAB) and Chromagar Candida® and incubated at 35 °C and 21 °C for seven days. In addition a SAB-broth was inoculated for enrichment. Identification of moulds was performed by examining macro- and micromorphology. In case identification was impossible using conventional methods, sequence analysis was suspended. In addition, the multiplex PCR Septifast (Roche, USA) was used for more rapid detection of certain *Candida species* and *Aspergillus fumigatus*. Aspergillus antigen was determined by using the serum galactomannan ELISA assay (Bio-Rad, Hercules, CA, USA) with a cut-off value for the optical density index of ≥0.5 for serum and ≥0.8 for BAL [9, 20]. For the identification of *Pneumocystis jirovecii* real-time PCR from BAL was performed [21, 22]. In some cases fungi were identified during autopsy or histological examination of tissues by direct microscopy by the department of pathology.

### IFD classification and study end-points

The primary end-point for this study was the one-year incidence for proven or probable invasive fungal diseases. IFDs were classified as probable or proven according to the revised definitions of the EORTC/MSG consensus group [9]. The secondary end-points for this study were the analysis of risk factors for IFD, the associated mortality and the impact of prophylaxis in preventing IFD.

### Statistical analysis

Statistical analysis was performed using SPSS 22 (IBM, Armonk US). All end-points were analysed using descriptive statistics: values were expressed as median and range for continuous variables and as percentages for categorical variables. Differences in patient’s characteristics were compared between groups with and without IFD and with and without aspergillosis. To explore possible risk factors for IFDs univariate and multivariate logistic regression analyses were performed. In addition, a sub-analysis for invasive aspergillosis was conducted excluding other IFDs. Risk factors with a *p*-value ≤ 0.05 in the univariate analysis were included in the multivariate model using the “Forward Wald method”. For the multivariate model a two-tailed *p*-value ≤ 0.05 was considered to be significant. Survival analysis was performed with Kaplan-Meier survival curves and the log-rank test.

## Results

### General characteristics of study population and of transplants

During the study period a total of 242 patients received 255 HSCTs – 11 patients had two and one patient 3 transplants. The median age was 46 years (range 19; 70 years) at the time of HSCT with 130 males (53.7 %) and 112 females (46.3 %). Underlying diseases were acute leukemia (71.9 %), chronic leukemia (6.2 %), lymphoma (11.2 %), myelodysplastic syndrome (4.1 %), aplastic anaemia (2.1 %), multiple myeloma (2.1 %) and other haematological diseases (2.4 %). Details concerning the EBMT risk score of patients, type of grafts, donors and conditioning regimens are included in Table 1.

**Table 1:** Possible risk factors for invasive fungal diseases (IFD, *n* = 28) and invasive aspergillosis (*n* = 18)

	Univariate analysis for IFDs						Univariate analysis for Aspergillosis					
Risk factors	No. of patients^a^	IFD		OR	95 % CI	*p*-value	No. of patients^a^	Aspergillus		OR	95 % CI	*p*-value
		*n*	%					*n*	%			
Gender												
male	130	10	7.7				126	6	4.8			
female	112	18	16.1	2.3	1.01–5.2	0.05	106	12	11.3	2.6	0.9–7.1	0.07
Underlying disease												
leukemia	189	22	11.6	1.0	0.4–2.7	0.9	179	12	6.7	0.6	0.2–1.6	0.3
lymphoma	27	5	18.5	1.9	0.7–5.5	0.2	27	5	18.5	3.4	1.1–10.3	0.03
others	26	1	3.8	-	-	-	26	1	3.8	-	-	-
Graft type												
PBSC	211	25	11.8	1.3	0.4–4.4	0.7	201	15	7.5	0.8	0.2–2.8	0.7
cord blood	22	3	13.6	1.2	0.3–4.5	0.8	22	3	13.6	2.1	0.5–7.7	0.3
bone marrow	9	0	0	-	-	-	9	0	0	-	-	-
Donor type												
unrelated	177	21	11.9				172	16	9			
related	65	7	10.8	0.9	0.4–2.2	0.8	60	2	3	0.3	0.1–1.5	0.2
HLA-matched donor												
matched	194	22	11.3				184	12	6.2			
mismatched	48	6	12.5	1.1	0.4–2.9	0.8	48	6	12.5	2.1	0.7–5.8	0.2
Conditioning regimen												
reduced-intensity	143	17	11.9				137	11	8			
myeloablative	99	11	11.1	0.9	0.4–2.1	0.9	95	7	7.4	0.9	0.3–2.4	0.9
EBMT score												
EBMT ≤ 2	69	13	18.8				64	8	12.5			
EBMT > 2	173	15	8.7	0.4	0.2–0.9	0.03	168	10	6	0.4	0.2–1.2	0.1
acute GvHD												
no GvHD or I-II°	167	18	10.8				158	9	5.7			
severe GvHD	75	10	13.3	1.3	0.6–2.9	0.6	74	9	12.2	2.3	0.9–6	0.09
chronic GvHD												
no GvHD or I-II°	188	22	11.7				181	15	8.3			
severe GvHD	54	6	11.1	0.9	0.4–2.5	0.9	51	3	5.9	0.7	0.2–2.5	0.6
Intensified GvHD therapy^b^												
no	215	19	8.8				206	10	4.9			
yes	27	9	33.3	5.2	2–13.1	0.001	26	8	30.8	8.7	3.1–24.8	<0.001
CMV reactivation												
no	154	15	9.7				147	8	5.4			
yes	88	13	14.8	1.6	0.7–3.6	0.2	85	10	11.8	2.3	0.9–6.1	0.09
TMA												
no	225	22	9.8				215	12	5.6			
yes	17	6	35.3	5	1.7–14.9	0.004	17	6	35.3	9.2	2.9–29.2	<0.001
St.p. IFD												
no	213	25	11.7				204	16	7.8			
yes	29	3	10.3	0.9	0.3–3.1	0.8	28	2	7.1	0.9	0.2–4.2	0.9
Antifungal prophylaxis^c^												
no	160	24	15				152	16	10			
yes	82	4	4.9	0.3	0.1–0.9	0.03	80	2	2.5	0.2	0.1–0.97	0.05

^a^number of patients: *n* = 242 for all IFDs and *n* = 232 for aspergillosis excluding all other IFDs

^b^Intensified GvHD therapy: high-dose glucocorticoid (≥1 mg/kg body weight) and either etanercept or basiliximab

^c^Antifungal prophylaxis: systemic prophylaxis during aplasia and during post-engraftment

Abbreviations: *OR* odds ratio, *CI* confidence interval, *PBSC* peripheral blood stem cells, *HLA* human leukocyte antigen, *EBMT* European Society of Blood and Marrow Transplantation, *GvHD* graft-versus-host disease, *CMV* cytomegalovirus, *TMA* transplant-associated microangiopathy

### Incidence and characteristics of IFDs

During the study period a total of 28 patients experienced an IFD (11.6 %), 25 of them (89.3 %) during the first year after HSCT. Therefore, the one-year incidence for IFD was 10.3 % (25/242), with peaks in the years 2010 (6/42, 14.3 %) and 2012 (6/44, 13.6 %), an average incidence in the years 2009 (5/53, 9.4 %) and 2013 (6/61, 9.8 %) and the lowest incidence in the year 2011 (2/42, 4.8 %).

Among IFDs invasive aspergillosis was predominant (18/28, 64.3 %) with 17 pulmonary and one cerebral aspergillosis. Second came invasive candidiasis (7/28, 25 %) and third pneumocystis pneumonia (3/28, 10.7 %). Eight IFDs (28.6 %) were proven infections and the other 20 IFDs (71.4 %) were classified as probable. Among the proven IFDs the following fungal species were identified: *Candida albicans* (*n* = 1), *Candida glabrata complex* (*n* = 2), *Candida krusei* (*n* = 1), *Candida species* (*n* = 1), *Aspergillus section fumigati* (*n* = 1) and *Aspergillus species* (*n* = 2). Pathogens of probable IFDs included *Candida albicans* (*n* = 2)*, Pneumocystis jirovecii* (*n* = 3)*, Aspergillus section fumigati* (*n* = 1)*, Aspergillus section nigri* (*n* = 1), *Aspergillus species* (*n* = 1) and 12 cases of invasive aspergillosis diagnosed on basis of a suspicious CT-scan and positive test for galactomannan antigen.

**Fig. 1:**
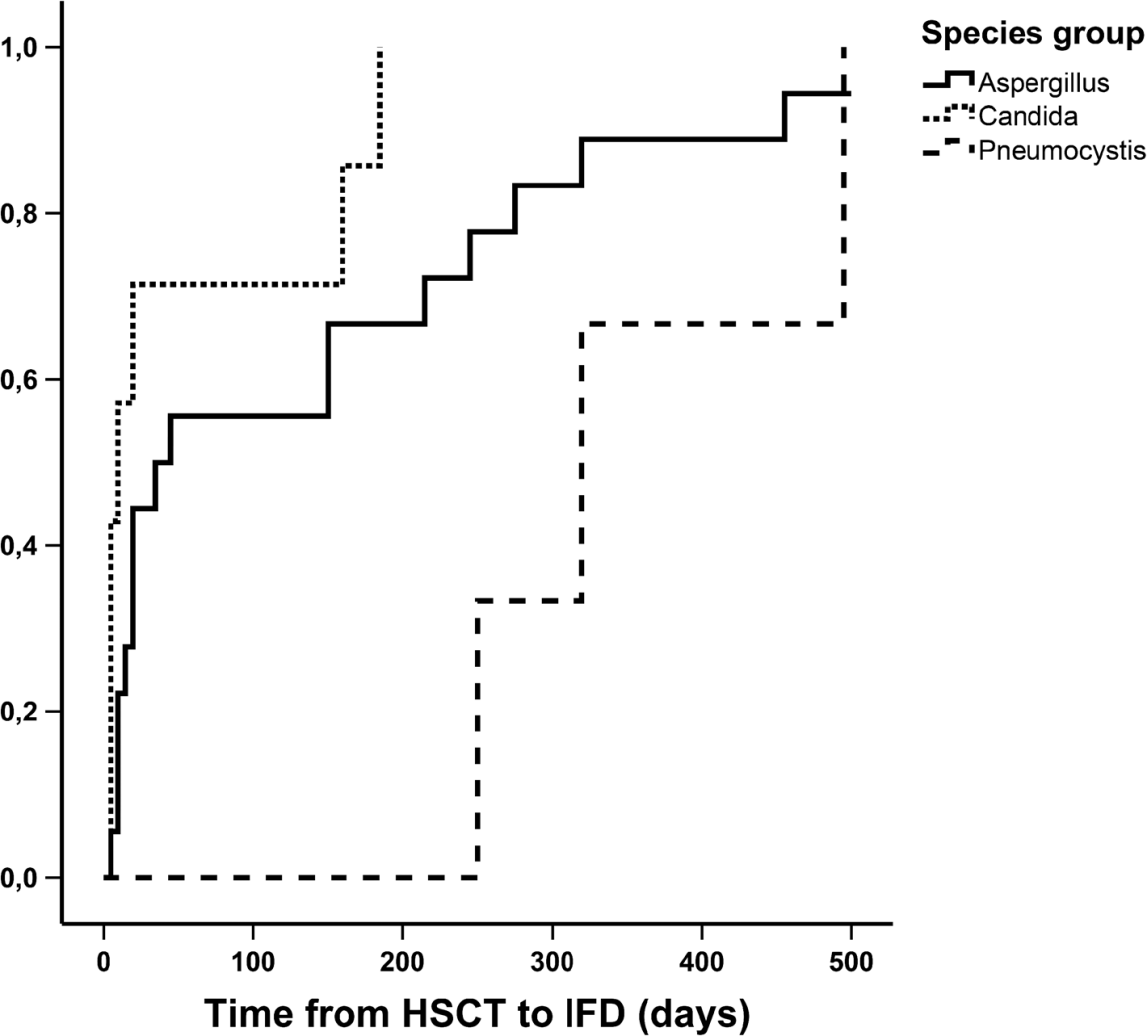
Time to diagnosis of invasive fungal disease (IFD) after allogeneic hematopoietic stem cell transplantation (HSCT). Invasive candidiasis (Candida, *n* = 7) occurred first (median day +8), followed by invasive aspergillosis (Aspergillus, *n* = 18, median day +36) and pneumocystis pneumonia (Pneumocystis, *n* = 3, median day +319)

As shown in Fig. 1, the median time from HSCT to the diagnosis of IFD was 8 days for invasive candidiasis, 36 days for invasive aspergillosis and 319 days for pneumocystis pneumonia. Twelve percent of patients (29/242) had already experienced a documented IFD before HSCT and three of those patients also developed an IFD after HSCT. But only in a single case a recurrence of a previous invasive aspergillosis can be assumed.

### Risk factors of IFD

The univariate logistic regression analyses for all IFDs as well as the sub-analysis for invasive aspergillosis are summarized in Table 1. Among the pre-transplant risk factors, only female sex was predictive for a higher risk for IFD (odds ratio (OR) 2.3, *p* = 0.05) and lymphoma as underlying disease was associated with a higher risk for invasive aspergillosis (OR 3.4, *p* = 0.03), whereas an EBMT-score of > 2 was associated with a lower risk for IFD (OR 0.4, *p* = 0.03). However, all three parameters did not reach significance (p ≤ 0.05) in the multivariate analysis. Intensified salvage therapy for severe GvHD (high-dose corticosteroids (≥1 mg/kg body weight) and basiliximab or etanercept) was a highly significant risk factor for IFD (OR 5.2, *p* = 0.001) and for invasive aspergillosis (OR 8.7, *p* < 0.001). Among the post-transplant complications transplant-associated microangiopathy (TMA) was significantly associated with IFD (OR 5.0, *p* = 0.004) and with invasive aspergillosis (OR 9.2, *p* < 0.001). Intensified GvHD therapy and TMA were moderately correlated risk factors (Spearman’s rho 0.3, *p* < 0.0001). Prophylaxis proved only to be significantly protective if it was given during aplasia as well as during post-engraftment (OR 0.3, *p* = 0.03 for IFD and OR 0.2, *p* = 0.05 for invasive aspergillosis). In the multivariate model only intensified GvHD therapy and TMA remained significantly associated with an increased risk for IFD and for invasive aspergillosis, while antifungal prophylaxis given during aplasia and post-engraftment was associated with a decreased risk (see Table 2).

**Table 2:** Multivariate logistic regression for all IFDs and for aspergillosis (significance level *p* ≤ 0.05)

Risk factors	AOR	all IFDs			Aspergillosis	
		95 % CI	p-value	AOR	95 % CI	p-value
Intensified GvHD therapy	3.6	1.3–9.9	0.01	5.4	1.7–17.3	0.01
TMA	3.7	1.1–12.9	0.04	6.7	1.7–26.3	0.01
Antifungal prophylaxis	0.3	0.1–0.8	0.02	0.2	0.03–0.9	0.03
Sex (female)	ns	ns	ns	x	x	x
EBMT score >2	ns	ns	ns	x	x	x
Lymphoma	x	x	x	ns	ns	ns

Abbreviations: *AOR* adjusted odds ratio, *CI* confidence interval, *IFD* invasive fungal disease, *GvHD* graft-versus-host disease, *TMA* transplant-associated microangiopathy, *EBMT* European Society for Blood and Marrow Transplantation, *ns* not significant, *x* not included in the model

### Outcome and mortality of patients with IFD

**Fig. 2:**
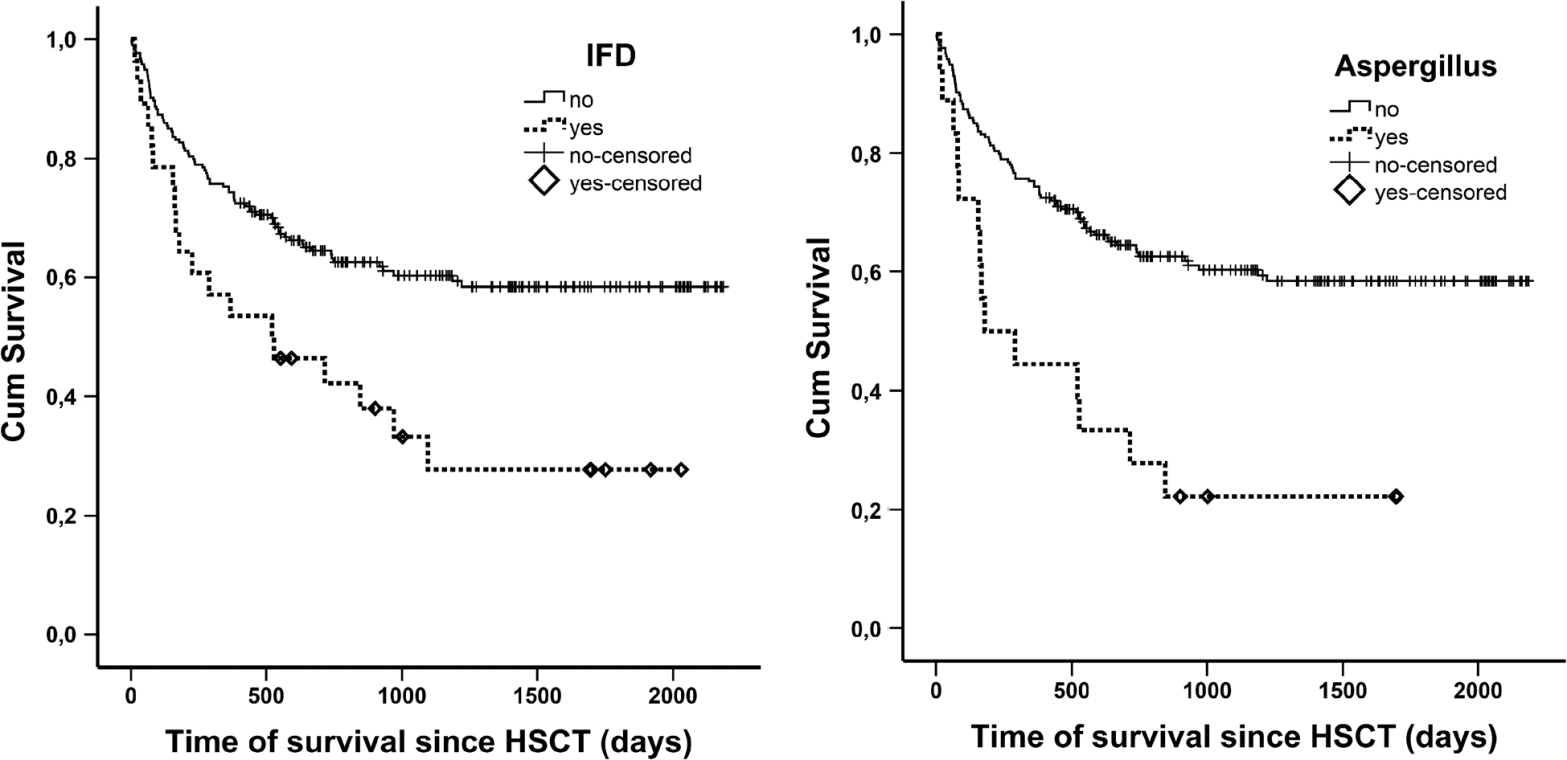
Cumulative survival curves of allogeneic hematopoietic stem cell transplant (HSCT) recipients. The survival curves compare patients without invasive fungal disease (IFD) to patients with IFD or with invasive aspergillosis during the follow-up period until December 31st 2014 showing that the survival in the group of patients with IFD (*n* = 28) or with Aspergillus (*n* = 18) was significantly worse (log rank test IFD vs. non-IFD *p* = 0.003 and aspergillosis vs. non-IFD *p* = 0.002) than in the group without IFD (*n* = 214)

The median survival during our study period was 677 days (range 2; 2185) for patients without IFD, 525 days for patients with IFD (range 14; 2030) and 235 days for patients with invasive aspergillosis (range 14; 1699) (see Fig. 2). Thus, the presence of an IFD was associated with a higher mortality rate compared to patients without IFD (48 % vs. 25.3 %, *p* = 0.02) during the first year after HSCT. The higher mortality rate was even more pronounced for patients with invasive aspergillosis (62.5 %, *p* = 0.003). Patients with IFD were also admitted to an ICU more often (18/28, 64.3 %) than patients without IFD (26/214, 12.1 %, *p* < 0.0001).

### Use of antifungal prophylaxis and therapy

Systemic antifungal prophylaxis was given to 43.3 % (105/242) of patients during aplasia, with the most commonly used antifungals being posaconazole (52/105, 49.5 %), voriconazole (28/105, 26.7 %) and fluconazole (17/105, 16.2 %). The use of prophylaxis during aplasia increased during our study period from 15.1 % in 2009 to 68.9 % in 2013. Sixty-five percent (158/242) of patients received prophylaxis during post-engraftment.

Thirty-six percent (88/242) of patients received empirical antifungal therapy during aplasia, with caspofungin (44/88, 50 %) as the first choice followed by liposomal amphotericin B (18/88, 20.5 %). In total, 69 % (167/242) of patients received an antifungal agent as either prophylaxis or empirical therapy during aplasia.

Of 25 IFD patients with either invasive aspergillosis or *Candida* infection, 56 % (14/25) were without prophylaxis at the time of IFD diagnosis. Sixteen percent (4/25) developed an invasive aspergillosis under fluconazole and 28 % (7/25) had mould-active prophylaxis but developed a fungal “breakthrough infection”. Of these 7 cases, 6 patients received posaconazole and suffered from invasive aspergillosis (*n* = 5) and invasive candidiasis (*n* = 1), and a single patient developed invasive candidiasis while receiving caspofungin. Overall, 5.3 % (6/113) of patients who received posaconazole prophylaxis at any time during aplasia or post-engraftment developed a “breakthrough” IFD compared to 6.3 % (4/64) of patients under fluconazole prophylaxis.

## Discussion

In the present single-centre study at our Bone Marrow Transplant Unit, we observed a one-year incidence of 10.3 % for IFDs after allogeneic HSCT during the years 2009–2013. Although the incidence rate did fluctuate, the overall trend appeared to be stable. Invasive aspergillosis was the predominant IFD constituting nearly two-thirds of IFDs. This epidemiological situation emphasises the increasing importance of mould infections over yeast infections. Different incidence rates have been reported – the TRANSNET group reported a low incidence of only 3.4 % for IFDs, with a higher rate in transplant situations using allogeneic mismatched related donors (8.1 %) or unrelated donors (7.7 %) [23]. The incidence rate at our centre was slightly higher than in other studies [13, 23]. However, considering the moderate use of antifungal prophylaxis, an incidence rate of 10.3 % appears to be quite low compared to centres with general use of prophylaxis [13]. IFDs and especially invasive aspergillosis were associated with a significantly higher mortality and a higher risk for admission to an intensive care unit. This again emphasises the importance of preventing IFDs in patients after HSCT to improve their overall survival.

Compared to other studies, we observed that IFDs occurred quite early after HSCT at our centre. The median time to IFD was only 36 days (8 days for candidiasis, 36 days for aspergillosis) while other studies reported a median time to IFD of 139 days [13] or 174 days [24] and the TRANSNET study observed that candidiasis and aspergillosis occurred after a median of 61 and 99 days, respectively [23]. In our cohort 15 IFDs occurred very early after HSCT (median of 9 days, range 1; 41), while all other IFDs occurred more than 150 days after HSCT. In the early IFD group only 33.3 % received prophylaxis during aplasia (fluconazole, *n* = 2; posaconazole, *n* = 3). In other centres all patients [13, 24] or the majority of patients [11] received at least fluconazole prophylaxis during aplasia. Limited use of fluconazole prophylaxis at our centre might explain the early onset of candidiasis [8], but not of invasive aspergillosis. Other factors like time to engraftment, conditioning regimen and donor grafts did not provide an explanation for the early incidence of IFDs in our cohort compared to other centres. Interestingly, Sun et al. described that matched sibling donors had a much later onset of IFDs (median of 142 days) than haploidentical donors (median of 23 days) [11]. Similarly, we observed that the median time to IFD was much earlier for mismatched unrelated donors (median of 14 days) and matched unrelated donors (median of 41 days) than for matched sibling donors (median of 156 days).

Among clinical risk factors only intensified GvHD therapy and TMA as post-transplant complications remained associated with IFD in the multivariate analysis. Severe GvHD is a known risk factor for IFD [1, 2] and an accepted indication for antifungal prophylaxis during post-engraftment [7]. Salvage therapy with high-dose corticosteroids and basiliximab or etanercept is used only in steroid-refractory GvHD with gastrointestinal involvement, therefore presenting a group with very high risk for IFD. Transplant-associated microangiopathy is a serious complication associated with the use of immunosuppressive agents like cyclosporine [25]. Both risk factors were moderately correlated and strongly associated with a high risk for IFDs. This shows that patients with very severe GvHD and need of intensified immunosuppressive treatment are also most vulnerable for IFDs.

The role of antifungal prophylaxis in preventing IFDs has been a topic of discussion during the last years. Although the effectiveness of fluconazole prophylaxis in preventing *Candida*infections has been proven more than twenty years ago [26], the shifting epidemiology often necessitates the use of regimens that also cover *Aspergillus species* [7, 27]. The setting of this study is interesting because of its moderate use of prophylaxis. The almost five-fold increase in use of prophylaxis during aplasia at our centre, apparently did not affect the incidence of IFDs. Another single-centre study from Western Austria reported an incidence of 13 % despite administering posaconazole to all patients during neutropenia and they reported an increasing rate of Mucorales and rare fungal pathogens [28]. At our centre we did not observe any mucormycoses or a shift towards rare pathogens. These findings draw attention to the fact that general use of prophylaxis might increase selection pressure in preference of non-Aspergillus moulds.

The overall rate of IFDs under systemic antifungal prophylaxis was 4.5 % (11/242). At our centre the rate of breakthrough infections under posaconazole (5.3 %) was similar to the study by Ullman et al. [4] who included only patients with GvHD. However, in the present study we did not routinely measure plasma concentrations of posaconazole and therefore inadequate blood levels could have been a possible reason for failure of antifungal prophylaxis. Nowadays, new formulations of posaconazole might improve absorption and avoid breakthrough infections [29], but this has yet to be established.

This study was conducted retrospectively and had therefore some limitations due to its design. Due to the use of galactomannan antigen and fungal PCR as diagnostic tools, proven mould infections were only rarely diagnosed as this would demand a biopsy which was often not feasible because of the high risk for complications in a large number of patients. The selection of patients receiving prophylaxis was due to the clinician’s evaluation, which is more difficult to analyze than objective criteria. A prospective design might eliminate these factors and present a clearer risk assessment.

One of the strengths of this study was the close follow-up of each patient during the first year after transplant. This allowed for a good evaluation of the one-year incidence of IFDs. The moderate use of prophylaxis, which differs from many other haematological centres, gave us an interesting background to assess the epidemiological trend of fungal infections.

## Conclusions

We documented a one-year incidence for IFDs of 10.3 % and no selection of rare pathogens at a centre with moderate use of antifungal prophylaxis. The predominant IFD was invasive aspergillosis and patients with IFD had a significantly higher mortality and an increased risk of requiring intensive care compared to HSCT recipients without IFD. Intensified immunosuppressive therapy and transplant-associated microangiopathy were significant risk factors for IFDs.
